# QT_c_ interval and ventricular action potential prolongation in the *Mecp2* ^*Null/*+^ murine model of Rett syndrome

**DOI:** 10.14814/phy2.15437

**Published:** 2022-10-05

**Authors:** Hongwei Cheng, Ian Charles, Andrew F. James, Ana P. Abdala, Jules C. Hancox

**Affiliations:** ^1^ School of Physiology, Pharmacology and Neuroscience University Walk Bristol UK

**Keywords:** action potential, APD_90_, eleclazine, GS‐6615, MECP2, QT interval, ranolazine, repolarization, Rett syndrome, RTT

## Abstract

Rett Syndrome (RTT) is a congenital, X‐chromosome‐linked developmental disorder characterized by developmental delay, dysautonomia, and breathing irregularities. RTT is also associated with sudden death and QT intervals are prolonged in some RTT patients. Most individuals with RTT have mutations in the *MECP2* gene. Whilst there is some evidence for QT prolongation in mouse models of RTT, there is comparatively little information on how loss of *Mecp2* function affects ventricular action potentials (APs) and, to‐date, none on ventricular APs from female RTT mice. Accordingly, the present study was conducted to determine ECG and ventricular AP characteristics of *Mecp2* ^*Null/+*^ female mice. ECG recordings from 12–13 month old female *Mecp2* ^*Null/+*^ mice showed prolonged rate corrected QT (QTc) intervals compared to wild‐type (WT) controls. Although *Mecp2* ^*Null/+*^ animals exhibited longer periods of apnoea than did controls, no correlation between apnoea length and QT_c_ interval was observed. Action potentials (APs) from *Mecp2* ^*Null/+*^ myocytes had longer APD_90_ values than those from WT myocytes and showed augmented triangulation. Application of the investigational I_Na,Late_ inhibitor GS‐6615 (eleclazine; 10 μM) reduced both APD_90_ and AP triangulation in *Mecp2* ^*Null/+*^ and WT myocytes. These results constitute the first direct demonstration of delayed repolarization in *Mecp2* ^*Null/+*^ myocytes and provide further evidence that GS‐6615 may have potential as an intervention against QT prolongation in RTT.

## Graphical abstract

QTc intervals and action potentials (APs) from ventricular myocytes were found to be prolonged in females from a murine model of Rett Syndrome (RTT). APs in ventricular myocytes from RTT mice were shortened by GS‐6615, a late sodium current inhibitor.

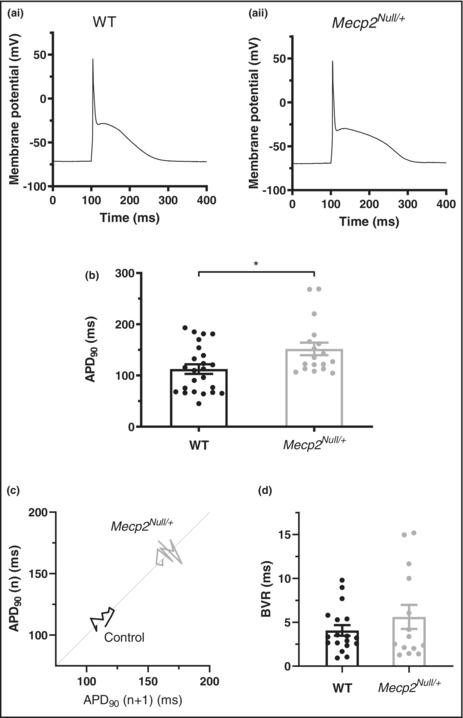

## 1 INTRODUCTION

Rett Syndrome (RTT) is a congenital X‐chromosome linked disorder that is characterized by neurodevelopmental delay, seizures, autonomic dysfunction and respiratory difficulties (Liyanage & Rastegar, 2014; Neul et al., 2010; Ramirez et al., 2013; Rett, 1969; Rett, 2016). Over 90% of patients with classic RTT have mutations to the *MECP2* gene, which encodes an X‐chromosome linked transcriptional regulator Methyl‐Cp‐binding protein 2 (Amir et al., 1999; Bienvenu et al., 2000; Huppke et al., 2000; Kim & Cook Jr., 2000; Liyanage & Rastegar, 2014; Neul et al., 2008; Neul et al., 2010). Due to this X‐linkage, males exhibit more severe disease phenotypes and most die within a year of birth (Liyanage & Rastegar, 2014); so the majority of RTT patients are female. RTT has an annual mortality rate of 1.2%, a little over a quarter of which is accounted for by sudden deaths (Kerr et al., 1997). Consistent with a potential cardiac contribution to sudden death in the syndrome, there is evidence that some RTT patients exhibit abnormalities in ventricular repolarization (Clark et al., 2020; Crosson et al., 2017; Ellaway et al., 1999; Guideri et al., 2001; McCauley et al., 2011; Sekul et al., 1994). It is now firmly established that a proportion of RTT patients show prolongation of the rate corrected QT (QTc) interval of the ECG, although the prevalence of QT_c_ prolongation differs between studies (from ~7 to 55%) (Clark et al., 2020; Crosson et al., 2017; Ellaway et al., 1999; Guideri et al., 2001; McCauley et al., 2011; Sekul et al., 1994). There is some evidence that particular *MECP2* mutations (R255*, T158M, or large deletions) are more likely to predispose to QT_c_ prolongation (Clark et al., 2020; Crosson et al., 2017). A primate (cynomolgous monkey) *MECP2* mutant model of RTT also exhibits prolongation of the QT_c_ interval (Chen et al., 2017).

A number of different mouse models have been developed that recapitulate major symptoms in RTT (for a review see Vashi & Justice, 2019). Amongst these, two *Mecp2*‐null models have been widely employed (Vashi & Justice, 2019). The *Mecp2* ^*tm1.1 Jae*^ line expresses small fragments of the MECP2 protein (Chen et al., 2001) and the *Mecp2* ^*tm1.1 Bird*^ line entirely lacks the MECP2 protein product (Guy et al., 2001). Mice from the ‘Bird’ strain develop progressive neurological and behavioral deficits that recapitulate those in human RTT and this model has been used extensively to study the underlying basis of RTT (Guy et al., 2001; Katz et al., 2012; Vashi & Justice, 2019). In 2011, McCauley et al published work using the ‘Bird’ strain that sheds light onto QT interval prolongation in RTT (McCauley et al., 2011). These authors reported QT prolongation in *Mecp2* ^*Null/Y*^ males of 2–3 months of age and in *Mecp2* ^*Null/+*^ females of 10 months of age (with younger females not exhibiting significant QT_c_ prolongation, highlighting a development‐dependence to this change; McCauley et al., 2011). RTT animals also showed an increased susceptibility to ventricular arrhythmia induced by programmed stimulation. Significantly, QT_c_ prolongation was also observed in animals in which *Mecp2* deletion was confined to the nervous system, indicating that the cardiac changes underlying this phenomenon are consequent upon changes that occur in the nervous system (McCauley et al., 2011). In the same study, additional experiments were conducted on isolated ventricular myocytes from *Mecp2* ^*Null/Y*^ males that showed an increased “late” sodium current, I_Na,Late_, in RTT compared to wild‐type (WT) myocytes (McCauley et al., 2011). The anti‐seizure drug phenytoin both decreased I_Na,Late_ and reduced QT_c_ interval and arrhythmia in *Mecp2* ^*Null/Y*^ males (McCauley et al., 2011). Some subsequent studies have also reported QT_c_ prolongation in RTT mice (Herrera et al., 2015; Mucerino et al., 2017), although one of these showed that phenytoin exacerbates breathing problems in RTT animals, which may limit its therapeutic value for shortening the QT_c_ interval (Herrera et al., 2015). In contrast to these studies, a different investigation of *Mecp2* ^*Null/Y*^ mice did not observe prolonged QT_c_ intervals in experiments performed at between 6 and 8 weeks of age (Hara et al., 2015).

Until recently, no study has investigated ventricular action potential (AP) repolarization per se in any RTT model. Very recently, however, the results of experiments on 2–3 month old *Mecp2* ^*Null/Y*^ males have confirmed the development of QT_c_ interval prolongation in the ‘Bird’ model; they also demonstrated that APs from ventricular myocytes isolated from RRT mouse hearts are prolonged (increased APD_90_) and exhibit increased triangulation and APD instability compared to WT controls (Cheng et al., 2022). Similar to the study by McCauley and colleagues, an increased I_Na,Late_ was found in *Mecp2* ^*Null/Y*^ compared to WT myocytes. I_Na,Late_ in *Mecp2* ^*Null/Y*^ myocytes retained sensitivity to the investigational I_Na,Late_ inhibitor GS‐6615 (also known as eleclazine), which was also found to abbreviate AP duration (Cheng et al., 2022). To date, no such study has been performed on *Mecp2* ^*Null/+*^ females. This is perhaps unsurprising given that in order for QT_c_ prolongation to be observed *Mecp2* ^*Null/+*^ females must be kept for at least 10 months, increasing the complexity and cost of such an undertaking. The slower development of a RTT phenotype in females than males is attributable to the fact that the *Mecp2* gene is located on the X chromosome (as is also the case in humans). Males possess only one copy of the gene and thus in *Mecp2* ^*Null/Y*^ animals the gene product is entirely absent; *Mecp2* ^*Null/+*^ females have one normal copy of the gene and so are heterozygous for ‘null’ protein. Comparable information in females to that for males would be valuable, given that the RTT patient population is predominantly female. Accordingly, this study was undertaken to characterize repolarization in 12–13 month old *Mecp2* ^*Null/+*^ females at the level of the ECG in intact mice and through measurement of APs from isolated ventricular myocytes.

## 2 METHODS

### 2.1 Mouse model of RTT employed in this study

All experiments were approved by the University of Bristol Animal Welfare Ethical Review Board (AWERB) and were carried out in accordance with UK Home Office legislation. The murine model of RTT employed for this investigation (the “Bird” strain (Guy et al., 2001)), had deletions of the third and fourth exons of *Mecp2*. Mice were genotyped as described previously (Cheng et al., 2022). McCauley et al reported no significant difference between QTc intervals in WT and *Mecp2* ^*Null/+*^ mice at 4–5 months of age, with differences becoming evident at older ages (10 months) (McCauley et al., 2011). Consequently, for this study all experiments were performed on female mice of 12–13 months of age. This age‐range was chosen to allow sufficient time for progressive changes to develop and thereby to optimize the likelihood of observing repolarization differences between WT and *Mecp2* ^*Null/+*^ strains.

### 2.2 Electrocardiogram (ECG) measurement

Mice were anesthetized by 1.5% isoflurane and ECG measurements were obtained 5 mins after anesthesia had been established. Surface lead II ECG measurements were obtained as described previously (Cheng et al., 2022). Transcutaneous needle electrodes were placed as follows: positive electrode in left hind leg; negative electrode in right front leg; ground electrode in right hind leg. Signals were high‐pass filtered at 10 Hz, with a low‐pass setting of 1 kHz. Measurements were made of RR interval (and from this heart rate); PR interval; QRS interval; QT and QT_c_ intervals. Mean values for each ECG parameter for each mouse were obtained from 5 consecutive ECG complexes, avoiding complexes upon which breathing noise was superimposed. The duration of the QT interval duration was taken as the time between onset of the QRS complex and time‐point after the T‐wave peak (Cheng et al., 2022; McCauley et al., 2011).

As previously (Cheng et al., 2022), two rate correction methods were employed in this investigation to obtain QT_c_ interval values (Equation 1: [Mitchell et al., 1998; Speerschneider & Thomsen, 2013; Cheng et al., 2022]; Equation 2: [Cheng et al., 2022; McCauley et al., 2011]):

(1) $${\mathrm{QT}}_{c}=\mathrm{QT}/{\left(\mathrm{RR}/100\right)}^{0.5}$$

(2) $${\mathrm{QT}}_{c}=\mathrm{QT}+0.3173\left(170-\mathrm{RR}\right)$$

### 2.3 Whole body plethysmography

Respiratory patterns were monitored using unrestrained whole‐body plethysmography (Emka Technologies, France) as described by Cheng et al. (2022). After a WT or *Mecp2* ^*Null/+*^ mouse had been placed in the recording chamber an adaption period of 20 mins was allowed; data for analysis were derived from a subsequent one‐hour recording period. Time‐series respiratory flow data were analyzed using a published custom analysis method in Spike 2 (V8.22, Cambridge Electronic Design, UK) (Abdala Sheikh, 2022). A running average of the total expiration time for each breath was taken every minute. If an expiration time was longer than 4 times this average, it was counted as an apnoea. Both apnoea count and length were recorded (Cheng et al., 2022).

### 2.4 Ventricular myocyte isolation

Animals were killed by cervical dislocation, the heart then excised and placed in ice‐cold isolation solution (composition given below) supplemented with 0.1 mM CaCl_2_ and 10 U/mL heparin. The heart was then cannulated and was Langendorff‐perfused for 3 minutes at 37°C at constant pressure of gravity (~80–100 cm H_2_O) with an isolation solution comprised of (in mM) 130 NaCl, 5.4 KCl, 0.4 NaH_2_PO_4_, 4.2 HEPES, 10 glucose, 1.4 MgCl_2_, 20 taurine, and 10 creatine (pH 7.4 with NaOH) (Cheng et al., 2022; Gadeberg et al., 2017). A 15‐minute period of perfusion with enzyme solution followed. Enzyme solution comprised of isolation solution to which were added 0.1 mM CaCl_2_, 0.07 mg/mL protease (Sigma, Type XIV), and 0.7 mg/mL collagenase (Worthington, Type 1). At the end of this period, the ventricles were removed from the Langendorff apparatus and were shaken in enzyme solution for 5 min before filtration and centrifugation. Ventricular myocytes were then resuspended in isolation solution plus 0.1 mM CaCl_2_ and stored at room temperature. Cells were used for up to 10 hours following myocyte isolation.

### 2.5 Action potential measurement

Ventricular myocytes were placed in a recording chamber (Cheng et al., 2022) and were superfused with a Tyrode's solution containing (in mM): 140 NaCl, 4 KCl, 1.5 CaCl_2_, 1 MgCl_2_, 10 glucose, 5 HEPES (pH 7.4 with NaOH). The superfusate temperature was 35‐37°C. A home‐built superfusion device allowed local superfusate to be rapidly (<1 s) exchanged (Levi et al., 1996). Patch pipettes were made from borosilicate glass (A‐M Systems Inc, Sequim, WA) pulled and fire polished to resistances of 2–3 MΩ (PP‐830 and MF83, Narishige, Japan). Pipettes were filled with a solution containing (in mM): 110 KCl, 10 NaCl, 0.4 MgCl_2_, 10 HEPES, 5 glucose, 5 K_2_ATP, 0.5 GTP‐Tris (pH 7.1 with KOH). Protocols were generated and data recorded online with pClamp 10 and a Digidata 1440A interface (Molecular Devices, USA). The digitization rate was 10 kHz; the signal was low‐pass filtered at 2 kHz. Action potentials (APs) were evoked at 1 second intervals by brief (3 ms) depolarizing current injection in membrane potential recording mode (Cheng et al., 2022). The threshold amplitude for these current pulses was monitored and is given in Results Table 2. Instability of repolarization was evaluated through measurement of beat‐to‐beat variability of AP repolarization (BVR). As previously (Cheng et al., 2022), this was quantified at 90% of AP repolarization (APD_90_) for 10–15 consecutive action potentials, using the equation:
(3)

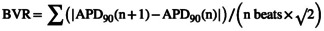




### 2.6 Data analysis and statistics

The numbers of ventricular myocytes and animals from which results were derived are given in the relevant Results text and accompanying Figure or Table Legends. Data are presented as mean ± SEM. Statistical comparisons utilized, as appropriate, a paired *t*‐test, unpaired *t*‐test with equal or unequal variances, and Mann–Whitney test. Statistical analysis was performed using Microsoft Excel (Microsoft Corporation, USA), Prism 8.4.3 (Graphpad Software Inc., USA) and Clampfit of pClamp 10.7 (Molecular Devices, USA). *p* < 0.05 was taken to be statistically significant. The data that support the findings of this study are available from the authors on reasonable request.

### 2.7 Ranolazine and GS‐6615 (eleclazine)

Ranolazine dihydrochloride was obtained from Sequoia Research Products Ltd, and 30 mM stock solution was made in distilled water. GS‐6615 was obtained from SYNthesis Med Chem, and 10 mM stock solution was made in DMSO. Stock solutions were diluted with standard Tyrode's solution to arrive at the final concentrations as given in the Results section.

## 3 RESULTS

### 3.1 ECG changes in *Mecp2* ^*Null*^ ^*/+*^ mice

**FIGURE 1:**
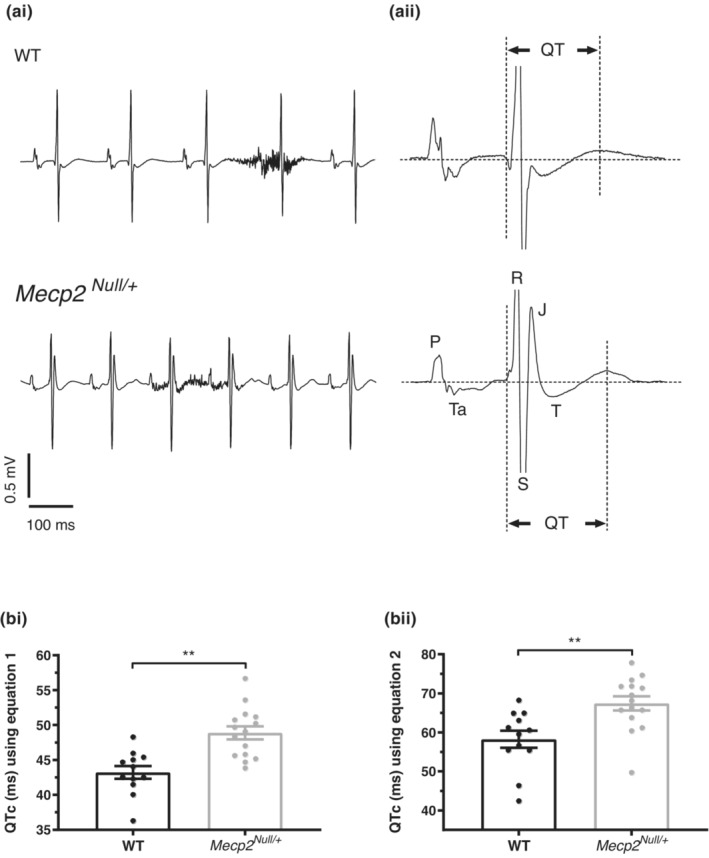
ECGs from WT and *Mecp2* ^*Null/+*^ animals. (a) Upper and lower panels of (ai) show ECG records from WT (upper) and *Mecp2* ^*Null/+*^ (lower) mice. The periods of high frequency noise in each trace represent breathing interference (aii) shows expanded single ECG cycles for WT and *Mecp2* ^*Null/+*^ animals (upper and lower, respectively), on which different portions of the ECG complex are marked and QT intervals indicated. (b) Bar chart plots show mean ± SEM values for QT_c_ intervals, with values from individual animals also plotted as circles (bi) shows comparison of QT_c_ interval values between 12 WT and 15 *Mecp2* ^*Null/+*^ mice calculated using equation 1 (Methods) and (bii) shows QT_c_ intervals for the same animals calculated using equation 2 (Methods). ** denotes *p* value of <0.01. Comparisons made using unpaired *t*‐test.

ECG measurements were compared between anesthetized female *Mecp2* ^*Null/+*^ and WT mice at 12–13 months of age. Figure 1a shows exemplar ECG traces from WT (upper panel) and *Mecp2* ^*Null/+*^ (lower panel) mice, with Figure 1ai showing series of successive ECG complexes and the QT interval illustrated for single ECG complexes from WT and *Mecp2* ^*Null/+*^ animals in Figure 1aii. Table 1 summarizes mean ECG data from 12 WT and 15 *Mecp2* ^*Null/+*^ animals. Heart rate was significantly greater in *Mecp2* ^*Null/+*^ (462.5 ± 21.4 bpm) than in WT mice (399.2 ± 18.5 bpm; *p* < 0.05); whilst RR interval was significantly smaller in *Mecp2* ^*Null/+*^ (134.9 ± 8.2 ms) than in WT mice (154.4 ± 8.3 ms, *p* < 0.05). PR interval was similar between the two strains. The QRS interval duration was numerically smaller in *Mecp2* ^*Null/+*^ than WT mice, but the difference between the two values was not statistically significant. The uncorrected QT interval duration was slightly longer (by 3 ms) in *Mecp2* ^*Null/+*^ than in WT mice, but the difference was not statistically significant. However, application of the two different rate correction methods to the QT interval (McCauley et al., 2011; Mitchell et al., 1998; Speerschneider & Thomsen, 2013; Cheng et al., 2022; see Methods) revealed a significant prolongation of QT_c_ interval (Figure 1b and Table 1). The two different correction formulae produced different absolute QT_c_ values, but with each method the mean QT_c_ interval of *Mecp2* ^*Null/+*^ mice was significantly greater than that of WT animals (by 5.7–9.2 ms; Table 1 and see Figure 1b for plotted mean and individual QT_c_ values). J‐waves were not uniformly observed; they were present in 8 out of 12 WT mice (absent in 4), and in 12 out of 15 *Mecp2* ^*Null/+*^ mice (absent in 3). The J‐wave amplitude was 0.22 ± 0.04 mV in 8 WT mice and 0.25 ± 0.03 mV in 12 *Mecp2* ^*Null/+*^ mice (*p* > 0.05 vs. WT; unpaired *t*‐test), meaning that there was no significant difference of J‐wave amplitude between WT and *Mecp2* ^*Null/+*^ mice in which these were present. Respiratory rates monitored during ECG measurement were 106.6 ± 9.9 and 122.4 ± 8.9 breaths per minute, respectively, from WT and *Mecp2* ^*Null/+*^ mice (*n* = 12 and 15 respectively; *p* > 0.2). These results demonstrate that at 12–13 months of age *Mecp2* ^*Null/+*^ mice have prolonged QT_c_ intervals compared to WT controls.

**TABLE 1:** ECG characteristics

Parameter	WT	*Mecp2* ^*Null/+*^	*t‐*test *p* value
RR (ms)	154.4 ± 8.3	134.9 ± 8.2	0.0281
HR (bpm)	399.2 ± 18.5	462.5 ± 21.4	0.0393
PR (ms)	46.1 ± 1.2	47.4 ± 2.3	0.6075
QRS (ms)	12.3 ± 0.5	11.8 ± 0.3	0.3897
QT (ms)	53.3 ± 1.0	56.3 ± 1.4	0.1102
QT_c_ (ms; equation 1)	43.2 ± 0.9	48.9 ± 0.9	0.0002
QT_c_ (ms; equation 2)	58.2 ± 2.2	67.4 ± 1.8	0.0031

*Note*: Mean ± SEM ECG parameters for 12 female wild‐type (WT) and 15 *Mecp2* ^*Null/+*^ mice. Statistical comparisons were made using unpaired *t*‐test, assuming unequal or equal variances as appropriate, and Mann–Whitney test.

**FIGURE 2:**
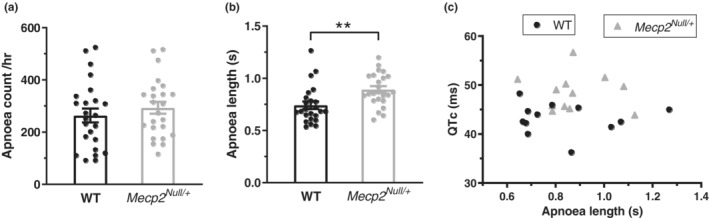
Analysis of apnoeas in WT and *Mecp2* ^*Null/+*^ mice. (a) Plot showing the mean apnoea counts observed for 24 WT and 24 *Mecp2* ^*Null/+*^ animals. These did not differ significantly from one another. An apnoea was determined to occur when the expiration time was longer than 4 times the average of the expiration time for each breath taken in the previous minute. An observation period of 1 h was used. (b) Plot showing the mean duration of each apnoea episode (apnoea length) for the same 24 WT and 24 *Mecp2* ^*Null/+*^ animals. **Represents *p* < 0.01; unpaired *t*‐test. (c) Plot of mean QT_c_ interval duration from 24 animals (12 WT and 12 *Mecp2* ^*Null/+*^, QT_c_ values calculated using equation 1) against apnoea length observed for the same animals. There was no significant correlation between the two parameters (*R* = 0.0239 and *p* = 0.9115).

Body plethysmography measurements were made separately from ECG measurements to evaluate periods of apnoea (Abdala et al., 2014; Cheng et al., 2022). Mean results (with superimposed data from measurements from individual animals) are shown in Figure 2. In all, measurements were made from 24 WT and 24 *Mecp2* ^*Null/+*^ animals. We observed no significant difference in the number of apnoea episodes in the two strains (Figure 2a). However, the mean duration of apnoea episodes was longer in *Mecp2* ^*Null/+*^ mice (Figure 2b). Figure 2c shows a plot of QT_c_ interval values (obtained using equation 1) against apnoea length. No significant correlation between the two values was found (*R* = 0.0239 and *p* = 0.9115).

### 3.2 AP changes in *Mecp2* ^*Null*^ ^*/+*^ mice

**FIGURE 3:**
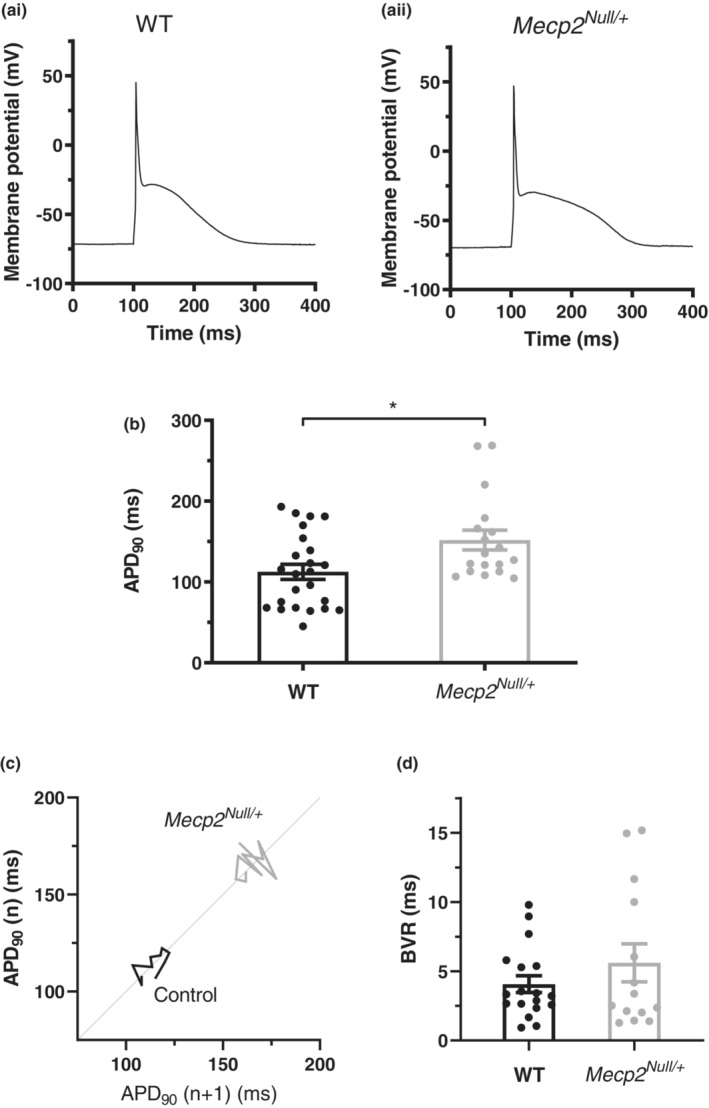
Action potentials (APs) from WT and *Mecp2* ^*Null/+*^ mice. (a) APs were elicited at a stimulation frequency of 1 Hz from ventricular myocytes isolated from WT (ai) and *Mecp2* ^*Null/+*^ (aii) mice. (b) Bar chart plots show mean APD_90_ values for WT and *Mecp2* ^*Null/+*^ myocytes (24 myocytes from 10 WT and 18 myocytes from 12 *Mecp2* ^*Null/+*^ animals). APD_90_ values from individual experiments are superimposed in each plot as circles. * denotes *p* < 0.05, unpaired *t* test. (c) Poincaré plot showing beat‐to‐beat variability (BVR) in APD_90_ for example WT and *Mecp2* ^*Null/+*^ myocytes over 10 successive APs. (d) Bar chart plots show mean values of BVR for WT and *Mecp2* ^*Null/+*^ myocytes (18 myocytes from 9 WT and 14 myocytes from 12 *Mecp2* ^*Null/+*^ animals). BVR values from individual experiments are superimposed in each plot as circles. BVR values were found not to significantly differ between WT and *Mecp2* ^*Null/+*^ myocytes (*p* > 0.05, Mann–Whitney test).

Isolated ventricular myocytes were stimulated at 1 Hz with fixed duration (3 ms) depolarizing current injection to elicit APs (see Methods and Cheng et al., 2022). Figure 3ai,ii show exemplar APs from WT and *Mecp2* ^*Null/+*^ myocytes, respectively. Table 2 summarizes mean AP data gathered from 24 myocytes from 10 WT mice and 18 myocytes from 12 *Mecp2* ^*Null/+*^ animals. Several notable differences were observed between myocytes from the two mouse strains. First, the threshold amplitude of the current stimulus required to elicit APs was significantly smaller in myocytes from *Mecp2* ^*Null/+*^ animals (see Table 2). Second, the resting membrane potential (RMP) was ~2.8 mV less negative in RTT myocytes (*p* < 0.05). A similar observation has been made for myocytes from male *Mecp2* ^*Null/Y*^ myocytes for which RMP was ~2.7 mV less negative than in WT control myocytes (Cheng et al., 2022). Neither of mean AP overshoot potential nor mean AP amplitude significantly differed between RTT and WT myocytes. However, mean AP upstroke velocity was smaller in RTT (122.5 ± 6.7 V.s^−1^) than WT myocytes (150.1 ± 6.4 V.s^−1^; *p* < 0.01). AP duration (APD) parameters were quantified at multiple time‐points as indicated in Table 2. No statistically significant differences in APD between the two strains were found at time points up to and including APD_50_ (duration at 50% of complete repolarization). However, significant differences in APD_75_ and APD_90_ were seen (see Table 2 and, for APD_90_ also Figure 3b; WT and RTT values of 112.6 ± 9.4 and 151.8 ± 12.2 ms, respectively; *p* < 0.05). The difference between APD_25_ and APD_90_ was measured as an index of AP triangulation and was observed to be significantly greater in RTT than WT myocytes (Table 2). In recent AP measurements from ventricular myocytes from *Mecp2* ^*Null/Y*^ males, APD_90_ instability was found to be greater than that for WT myocytes (APD_90_ BVR; [Cheng et al., 2022]). This is a significant because increased AP instability is a pro‐arrhythmic marker (Hondeghem, 2007; Hondeghem et al., 2001). Figure 3c contains a Poincaré plot that shows examples of beat‐to‐beat variability of APD_90_ for WT and *Mecp2* ^*Null/+*^ APs. Figure 3d shows mean and individual BVR values calculated using equation 3 (Methods) for WT and *Mecp2* ^*Null/+*^ APs. Although there was marked overlap in the values of BVR recorded from the two groups, the largest three values were from the *Mecp2* ^*Null/+*^ group (5.6 ± 1.4 ms from 14 myocytes from 12 *Mecp2* ^*Null/+*^ mice; 4.1 ± 0.6 ms from 18 myocytes from 9 WT mice; *p* > 0.05, Mann–Whitney test).

**TABLE 2:** Ventricular action potential (AP) parameters

Parameter	WT	*Mecp2* ^*Null/+*^
Resting potential (mV)	−72.8 ± 0.8	−70.0 ± 1.0 *
Overshoot (mV)	43.6 ± 1.9	40.7 ± 3.1
Amplitude (mV)	116.4 ± 2.0	110.7 ± 3.6
V_max_ (V s^−1^)	150.1 ± 6.4	122.5 ± 6.7**
APD_10_ (ms)	0.4 ± 0.0	0.6 ± 0.1
APD_25_ (ms)	1.6 ± 0.2	2.2 ± 0.5
APD_50_ (ms)	5.2 ± 0.7	6.8 ± 1.4
APD_75_ (ms)	65.5 ± 7.5	94.0 ± 10.4*
APD_90_ (ms)	112.6 ± 9.4	151.8 ± 12.2*
APD_90_ ‐ APD_25_ (ms)	111.0 ± 9.3	149.7 ± 12.3*
Threshold stimulus (pA)	707.3 ± 45.6	576.4 ± 41.1*

*Note*: Mean ± SEM AP parameter values for APs recorded from isolated ventricular myocytes. 24 cells from 10 WT mice and 18 cells from 12 *Mecp2* ^*Null/+*^ mice. APs were elicited by 3 ms duration depolarizing current pulses applied at a stimulation frequency of 1 Hz. Threshold values are included in the table. * denotes *p* < 0.05 and ** denotes *p* < 0.01 from unpaired *t‐*test assuming equal or unequal variances, as appropriate.

### 3.3 Effects of GS‐6615 on *Mecp2* ^*Null*^ ^*/+*^ APs

**FIGURE 4:**
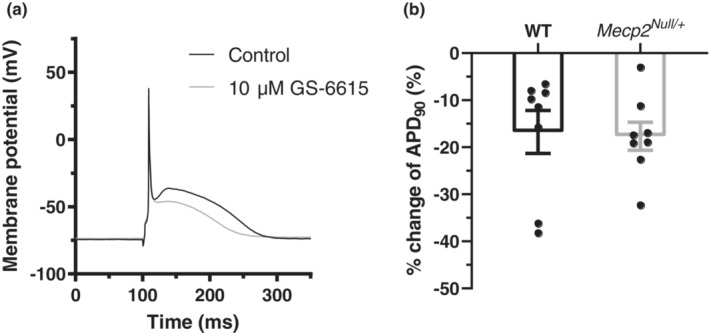
Effect of GS‐6615 on ventricular APs. (a) Example APs in control and 10 μM GS‐6615 for a *Mecp2* ^*Null/+*^ myocyte (AP stimulation frequency was 1 Hz). (b) Plots showing mean % change in AP duration with GS‐6615 at 90% repolarization (APD_90_) for each of WT and *Mecp2* ^*Null/+*^ conditions. Plots show data from 8 myocytes from 5 WT mice and 8 myocytes from 6 *Mecp2* ^*Null/+*^ mice. There was no significant difference between the magnitude of response between WT and *Mecp2* ^*Null/*Y^: GS‐6615 abbreviated APD_90_.

GS‐6615 (eleclazine) is an I_Na,Late_ inhibitor that has effectiveness against LQT3 Na channel mutations (El‐Bizri et al., 2018). Recent data from *Mecp2* ^*Null/Y*^ AP recordings is suggestive that GS‐6615 retains effectiveness and abbreviates APD_90_ in the RTT setting. To determine whether this also applies to female RTT mice APs, we applied 10 μM GS‐6615. Figure 4a shows *Mecp2* ^*Null/+*^ APs in control Tyrode's solution and following exposure to GS‐6615; AP abbreviation was observed. Figure 4b shows mean (and superimposed individual experiment) data showing the percentage reduction in APD_90_ observed for *Mecp2* ^*Null/+*^ myocyte APs and comparable data from WT myocytes, showing that GS‐6615 retained effectiveness in the RTT setting. GS‐6615 also decreased AP triangulation in the RTT setting (from 134.1 ± 19.6 ms to 110.2 ± 11.0 ms; *n* = 8 myocytes from 6 *Mecp2* ^*Null/+*^ mice; *p* < 0.05, paired *t*‐test). AP triangulation in WT myocytes was also decreased (from 109.0 ± 18.4 ms to 90.5 ± 16.6 ms; *n* = 8 myocytes from 5 WT mice; *p* < 0.05, paired *t*‐test). Similar experiments were also performed with the lignocaine relative ranolazine; however, as reported recently in experiments on male mouse WT and RTT myocytes (Cheng et al., 2022), ranolazine prolonged rather than abbreviated APD (data not shown).

## 4 DISCUSSION

### 4.1 Results in context

This is the first study to report lengthening of cardiac APs from a female RTT model of any species and only the second in a RTT model from either sex (Cheng et al., 2022). The Bazett's corrected QT (QT_c_) interval of female cynomolgus monkeys with MECP2 knockout is prolonged compared to controls (Chen et al., 2017), but further cardiac electrophysiological characterization of this model has not been published. In contrast with this simian model, mouse RTT models do not exhibit embryonic lethality for males, with significant male survival and with heterozygous female mice developing symptoms more slowly than in humans; marked deficits become visible in adulthood (Novarino, 2017; Vashi & Justice, 2019). In the original study that reported QT_c_ prolongation in RTT mice, female *Mecp2* ^*+/−*^ mice of 4 months of age showed no significant alterations in ECG parameters, whilst males of 2–3 months of age showed marked QT and QT_c_ prolongation and an increase in QRS width (with QT_c_ intervals of 53.7 ms and 67.6 ms in WT and *Mecp2* ^*Null/Y*^ animals, respectively [a 13.9 ms difference]; [McCauley et al., 2011]). Qualitatively similar changes to these ECG parameters were observed in older females, with mean QT_c_ intervals in WT and *Mecp2* ^*+/−*^ animals at 10 months of 50.3 and 58.1 ms, respectively (a 7.8 ms difference). A separate study attempted to group 11 month‐old *Mecp2* ^*+/−*^ animals into groups with and without QT_c_ prolongation (Mucerino et al., 2017), without any significant differences evident in RR interval between WT and *Mecp2* ^*+/−*^ animals. In the present study a clear pattern of QT_c_ prolongation in 12–13 month *Mecp2* ^*Null/+*^ animals was seen (Figure 1bi,ii). In our previous investigation of *Mecp2* ^*Null/Y*^ animals, we observed significant increases in QT and QT_c_ interval without significant changes in heart rate; QRS interval width also increased—observations that are similar to the original findings of McCauley et al (McCauley et al., 2011). In the older females investigated in this study, a significant increase in heart rate was seen (Table 1) and, perhaps due to this no significant changes in QRS width or uncorrected QT interval were observed, though marked QT_c_ prolongation (i.e., following rate correction) was found (Table 1).

The direct measurement of ventricular APs has the dual advantages of (i) control of stimulation rate and (ii) direct measurement of membrane potential. The same recording conditions and stimulation rate (1 Hz) were used here for female *Mecp2* ^*Null/+*^ myocytes as in our recent report that included AP data from myocytes from *Mecp2* ^*Null/Y*^ male animals from the same RTT model. It is instructive, therefore, to compare the differences in AP parameters between WT females observed in this study with those seen in males under identical experimental conditions (Cheng et al., 2022). In ventricular myocytes from 8–10 week *Mecp2* ^*Null/Y*^ animals, AP durations at 75% and 90% of complete repolarization were increased in *Mecp2* ^*Null/Y*^ compared to WT myocytes (with APD_90_ prolonged by ~50 ms); AP triangulation (APD_90_‐APD_25_) was also increased by ~50 ms in *Mecp2* ^*Null/Y*^ myocytes (Cheng et al., 2022). The present study shows that these parameters also differ between WT and *Mecp2* ^*Null/+*^ myocytes, with both APD_90_ and AP triangulation greater by ~39 ms in *Mecp2* ^*Null/+*^ than in WT control myocytes. The modest depolarization in resting potential and decrease in current required to elicit APs (Table 2) from *Mecp2* ^*Null/+*^ myocytes were also seen in myocytes from *Mecp2* ^*Null/Y*^ compared to those from WT controls (Cheng et al., 2022). Two differences from prior data on male myocytes were seen, however. First, AP upstroke velocity (V_max_) was significantly reduced in *Mecp2* ^*Null/+*^ compared to WT control myocytes (Table 2); in *Mecp2* ^*Null/Y*^ myocytes there was a tendency toward a reduction in V_max_, but this was not statistically significant (Cheng et al., 2022). Second, AP instability (BVR) was significantly greater in male RTT than control myocytes; here there was a trend toward an increase in BVR, but this did not reach statistical significance (Figure 3c,d). The overall similarity between AP observations from male and female animals is consistent with common underlying changes in the model. The differences between females and males in the extent of APD changes and in the statistical significance or otherwise of V_max_ and BVR alterations are consistent both with male–female differences in the time‐course of overall RTT phenotype development and, potentially, also with variation in the time‐courses over which changes to different electrophysiological parameters develop. We do not exclude the possibility that the differences seen here between WT and RTT myocyte AP parameters may be smaller at higher stimulation rates than the 1 Hz used here. Nevertheless, it is significant in this regard that delayed repolarization was observed in both AP and ECG measurements, which were taken under different recording conditions.

### 4.2 Consideration of the basis for altered repolarization in RTT mice

The need to keep females for at least 10 months (McCauley et al., 2011) to 12 or more months (this study) for repolarization abnormalities to be evident poses logistical and cost problems for the detailed interrogation of cellular electrophysiology in this model. With the exception of the AP recordings in the present study, the available cellular electrophysiology data in this RTT model come from experiments on myocytes from younger males (Cheng et al., 2022; McCauley et al., 2011). Male *Mecp2* ^*Null/Y*^ myocytes exhibit increased I_Na,Late_ (Cheng et al., 2022; McCauley et al., 2011) and a reduced fast I_Na_ magnitude (Cheng et al., 2022) compared to myocytes from WT controls. Such changes are commensurate both with a reduction in AP upstroke velocity and APD prolongation. The augmented I_Na,Late_ in male RTT myocytes was not accounted for by altered “window” current for I_Na_, as that was found to be smaller rather than augmented in *Mecp2* ^*Null/Y*^ myocytes (Cheng et al., 2022). As highlighted in considering the male AP data previously (Cheng et al., 2022), the modest depolarization in RMP and reduction in current required to elicit APs are suggestive of additional changes to (a) conductance(s) at the RMP, the underlying basis for which is not known at the present time (Cheng et al., 2022).

There was a lack of observable correlation between QT_c_ prolongation and apnoea duration in *Mecp2* ^*Null/+*^ animals (Figure 2), which is inconsistent with repolarization delay being a direct consequence of breathing abnormalities (this contrasts with obstructive sleep apnoea which can lead to QT_c_ prolongation [e.g., Walker et al., 2020; Sillanmäki et al., 2022]). This is also the case for male mice from this model (Cheng et al., 2022). McCauley et al showed that similar changes to repolarization and I_Na,Late_ were seen with both global *Mecp2* knockout and with knockout selective to the nervous system (McCauley et al., 2011). Thus, cardiac changes in this model are secondary to those in the nervous system, but once established can persist in isolated myocytes that are no longer under direct nervous system control. Data from the present study do not address the mechanism(s) underlying these changes. However, Herrera et al have shed additional light on arrhythmogenesis in the model (Herrera et al., 2016). They observed that death in this model was associated with spontaneous arrhythmias and conduction block; atropine mitigated these effects, suggestive of parasympathetic over‐activity. Mice were generated with cholinergic neurone specific *Mecp2* deletion and these exhibited QT_c_ prolongation and increased susceptibility to induced arrhythmias (Herrera et al., 2016). Restoration of *Mecp2* in cholinergic neurones rescued the cardiac phenotype (Herrera et al., 2016). Herrera et al discussed the possibility that changes in parasympathetic tone may be related to seizures (Herrera et al., 2016). Direct comparison with the present study (in which mean heart rate was not decreased) is difficult, however, because ECGs were monitored by telemetry (Herrera et al., 2016), whereas in the present study ECGs were measured from anesthetized animals. Additionally, the mechanism(s) that link altered nervous system activity to changes in cardiac electrophysiology that can persist in the absence of acute neural modulation remain(s) to be elucidated.

### 4.3 Clinical relevance

The direct measurement of ventricular AP prolongation in female *Mecp2* ^*Null/+*^ myocytes seen in this study together with prior data from *Mecp2* ^*Null/Y*^ male myocytes (Cheng et al., 2022; McCauley et al., 2011) provide an explanation for QT_c_ prolongation observed in RTT patients. Acute administration of the β adrenoceptor inhibitor propranolol to both WT and male *Mecp2* ^*Null/Y*^ mice failed to reduce QT_c_ interval and it failed also to protect *Mecp2* ^*Null/Y*^ mice subject from arrhythmias provoked by programmed electrical stimulation (McCauley et al., 2011). In contrast, the anticonvulsant agent, phenytoin, reduced QT_c_ interval and ventricular tachycardia in RTT mice and also reduced I_Na,Late_ in *Mecp2* ^*Null/Y*^ myocytes (McCauley et al., 2011). In a follow‐on study, chronic administration of propranolol had no effect on QT_c_ interval or arrhythmia susceptibility both in young *Mecp2* ^*Null/Y*^ males and 10‐month‐old *Mecp2* ^*Null/+*^ females (Herrera et al., 2015). By contrast chronic phenytoin corrected the prolonged QT_c_ interval and decreased ventricular arrhythmia susceptibility in *Mecp2* ^*Null/Y*^ males and 10‐month‐old *Mecp2* ^*Null/+*^ females (Herrera et al., 2015). Unfortunately, however, chronic phenytoin also worsened breathing patterns in RTT mice (Herrera et al., 2015). Retrospective analysis in the same study of data from the RTT Natural History Study identified 68 individuals with ECG measurements before drug treatment and who had received either propranolol or anti‐epileptic drugs with Na^+^ channel‐blocking properties. Numbers were insufficient to evaluate effects of β‐blocking drugs on the QT_c_ interval. However, of 64 individuals with multiple ECGs, 10 had a prolonged QT_c_ interval (> 450 ms) prior to anti‐epileptic drug treatment; 7 of 10 had QT_c_ values below the 450 ms threshold for QT_c_ prolongation after anti‐epileptic drug treatment (Herrera et al., 2015). These observations were suggestive that drugs with Na^+^ channel‐blocking activity are likely to be beneficial in RTT patients with QT_c_ prolongation, though none of the anti‐epileptic drugs other than phenytoin were tested in the RTT mouse model.

We have recently shown that acute application of GS‐6615 (eleclazine) and ranolazine (which is structurally related to lignocaine [Hancox & Doggrell, 2010]) reduce I_Na,Late_ from male *Mecp2* ^*Null/Y*^ myocytes; GS‐6615 also reduced APD_90_ and AP triangulation in *Mecp2* ^*Null/Y*^ myocytes (Cheng et al., 2022). The data shown in Figure 4 of this study indicate that GS‐6615, at the same concentration as applied previously to male myocytes, exhibits the ability to shorten APD_90_ and reduces triangulation in female *Mecp2* ^*Null/+*^ ventricular myocytes. This raises the possibility that GS‐6615 could have potential value for treatment of prolonged QT_c_ intervals in RTT patients. GS‐6615 is an investigational drug; however, it would be valuable to identify further agents that could be used to abbreviate repolarization. Ranolazine produces a prolongation of murine ventricular APs (Cheng et al., 2022; Lowe et al., 2012) and so it is difficult to establish its potential utility against QT_c_ prolongation in RTT using a murine model. European Society of Cardiology (ESC) guidelines for the treatment of ventricular arrhythmias and prevention of sudden death note that because both mexiletine and flecainide (class IB and IC antiarrhythmic drugs, respectively) can inhibit both fast I_Na_ and I_Na,Late_ (Hézsco et al., 2021; Nagatomo et al., 2000); they may have utility in the treatment of congenital long QT variant 3 (LQT3) (Priori et al., 2015). It would be valuable to establish effects of both drugs on QT_c_ interval and AP characteristics of this RTT model. Similarly, systematic investigation of Na^+^ channel blocking anti‐epileptic drugs other than phenytoin (Herrera et al., 2015) on repolarization in this model would be useful, particularly given their existing use to treat seizures in RTT.

## 5 CONCLUSIONS

The results of this study are consistent with earlier data showing QT_c_ interval prolongation in this model of RTT (Cheng et al., 2022; Herrera et al., 2015; Herrera et al., 2016; McCauley et al., 2011). The direct measurement of APs from *Mecp2* ^*Null/+*^ ventricular myocytes has also demonstrated delayed repolarization and showed results that are similar, albeit not identical, to those from younger *Mecp2* ^*Null/Y*^ male myocytes (Cheng et al., 2022). The beneficial effects of GS‐6615 in reducing APD_90_ and AP triangulation are similar to those reported recently in myocytes from male RTT mice and support the further preclinical investigation of this as a potential antiarrhythmic strategy in RTT. Additional investigation of existing (class IB and IC) anti‐arrhythmic and Na^+^ blocking anticonvulsant agents on repolarization in the model would also be desirable. Finally, further mechanistic work is required to determine the signaling pathway(s) that mediate changes in ventricular repolarization secondary to nervous system changes in the model. The earlier development of repolarization delay in males may make such an investigation more easily approachable, at least in the first instance, using *Mecp2* ^*Null/Y*^ males from this model.

## AUTHOR CONTRIBUTIONS

Conceptualization: JCH and APA; Funding acquisition: JCH, APA, AFJ; Supervision: JCH, APA, AFJ; Experimental Design: JCH, APA, AFJ, HC; Data acquisition and analysis: HC and IC; Manuscript drafting: JCH, APA, HC, AFJ, IC.

## CONFLICT OF INTEREST

The authors declare no conflict of interest.
